# To Treat or Not to Treat Bees? Handy VarLoad: A Predictive Model for *Varroa destructor* Load

**DOI:** 10.3390/pathogens10060678

**Published:** 2021-05-30

**Authors:** Hélène Dechatre, Lucie Michel, Samuel Soubeyrand, Alban Maisonnasse, Pierre Moreau, Yannick Poquet, Maryline Pioz, Cyril Vidau, Benjamin Basso, Fanny Mondet, André Kretzschmar

**Affiliations:** 1 INRAE (Institut National de la Rechernche Agronomique et de l’Environnement), Abeilles et Environnement, 84914 Avignon, France; helene.dechatre@gmail.com (H.D.); yannick.poquet@hotmail.com (Y.P.); maryline.pioz@inrae.fr (M.P.); benjamin.basso@inrae.fr (B.B.); 2 UMT PrADE (Unité Mixte Technologique: Protection de l’Abeille dans l’Environnement), 84914 Avignon, France; a.maisonnasse.adapi@free.fr (A.M.); cyril.vidau@itsap.asso.fr (C.V.); 3 INRAE (Institut National de la Rechernche Agronomique et de l’Environnement), BioSP, 84914 Avignon, France; lucie.michel@inrae.fr (L.M.); samuel.soubeyrand@inrae.fr (S.S.); 4 ADAPI (Association Pour le Développement de l’Apiculture en Provence), 13626 Aix en Provence, France; 5 Beekeeper, 07270 Empurany, France; pierre-pm.moreau@laposte.net; 6 ITSAP (Institut Technique et Scientifique de l’Abeille et de la Pollinisation), Institut de l’Abeille, 84914 Avignon, France

**Keywords:** *Apis mellifera*, *Varroa destructor*, treatment, predictive model, beekeeping, decision-making tool

## Abstract

The parasitic *Varroa destructor* is considered a major pathogenic threat to honey bees and to beekeeping. Without regular treatment against this mite, honey bee colonies can collapse within a 2–3-year period in temperate climates. Beyond this dramatic scenario, Varroa induces reductions in colony performance, which can have significant economic impacts for beekeepers. Unfortunately, until now, it has not been possible to predict the summer Varroa population size from its initial load in early spring. Here, we present models that use the Varroa load observed in the spring to predict the Varroa load one or three months later by using easily and quickly measurable data: phoretic Varroa load and capped brood cell numbers. Built on 1030 commercial colonies located in three regions in the south of France and sampled over a three-year period, these predictive models are tools designed to help professional beekeepers’ decision making regarding treatments against Varroa. Using these models, beekeepers will either be able to evaluate the risks and benefits of treating against Varroa or to anticipate the reduction in colony performance due to the mite during the beekeeping season.

## 1. Introduction

The parasite *Varroa destructor* is considered a major pathogenic threat to honey bees [1] and to beekeeping. This mite is an ectoparasite affecting both adult bees and broods. Female mites have two distinct stages: a phoretic stage on adult bees and a reproductive stage, which takes place inside a capped brood during bee metamorphosis. The Varroa threat is not new for the beekeeping community, but with colony importations and the commerce of bees, this threat continues to increase. Indeed, these circumstances favor the Varroa spread throughout territories and the world’s apiaries. This threat is all the more important given that the parasite spread is rapid [2]. Thus, bees and beekeepers cannot adapt and respond efficiently; on the contrary, Varroa, with continuous exposure to miticide treatments, responds with mechanisms of resistance [3,4,5]. Consequently, the current challenge is to develop new methods to limit Varroa numbers inside colonies. Without regular efficient treatment against this mite, honey bee colonies can collapse within a 2–3-year period in temperate climates. Varroa feeding on pupal hemolymph can induce a decrease in adult bee body weight and malformations as well as reducing their life spans, thus weakening their immune systems [6,7,8]. Thus, it seems logical that infested colonies are less productive and efficient than healthy colonies, which can have significant economic impacts for beekeepers [9,10]. Beyond a threshold of 3 phoretic Varroa mites per 100 bees, the decrease in performance is correlated with the Varroa load [10]. According to this study, a colony with more than 3 phoretic Varroa mites per 100 bees produces, on average, 2.65 kg less honey than a colony below this threshold. Unfortunately, until now, it has not been possible to predict, from the mite population size in the spring, the population load in the summer, despite studies by Arechavaleta-Velasco and Guzman-Novoa (2001), Harris et al. (2003), and Lodesani et al. (2002), confirmed significant correlations between the amount of brood and/or the fertility of the mites [11,12,13] and population growth [1].

Models of Varroa dynamics have been previously established but mainly carried theoretical descriptions and only allowed for the evaluation of the instantaneous Varroa load. Wilkinson and Smith’s model [14] was built from virtual colonies, and DeGrandi-Hoffman and Curry’s model [15] was based on the BEEPOP honey bee colony population dynamics model [16]; a BEEHAVE Varroa unit was developed by Becher et al. [17]. These models were based on parametric values available from previous studies [16,18,19,20,21,22,23,24,25,26]. As these models primarily work with mathematical extrapolation, instead of being data-derived, we assumed that the resulting parametric values could be revised. Additionally, in the twenty years since these models were published, Varroa biology may have coevolved with its host. The coevolution between Varroa and honey bees has been reported by Kurze et al. [27] and includes host resistance behaviors, which involve a decrease in the Varroa reproduction rate as well as perturbations in the biological cycle of the mite. Moreover, previous studies serving as the basis for model construction were based on honey bees with different European origins and on Africanized honey bees [19]. Honey bee origins affect Varroa reproduction [28] and, consequently, Varroa population sizes. To increase its predictability, here, we used a model based on empirical data.

The most important information for a beekeeper is not the Varroa load at the time of honey flow because most treatment compounds, even some labeled “natural” (e.g., formic acid or thymol), are banned or not recommended during honey flow [29]. The aim of this study was therefore to predict the Varroa load one or three months later, from its baseline level in early spring, to anticipate colony performance for honey flow, knowing that the reduced performance threshold is 3 phoretic Varroa mites per 100 bees. Aimed as a useful tool for beekeepers, the model Handy Varload is based on inexpensive, accessible, and quickly measurable data in the field.

## 2. Results

### 2.1. Variable Selection (for Variable Definitions, See Materials and Methods, Statistical Analysis)

The variable “phoretic Varroa” measured at *t* = 0 was continuous with 25% of zeros, 27% of the data in [0, 1], and 48% of the data in [1,30]. The zero-inflated beta distribution is similar to the beta distribution but allows zeros as response values in which the ν parameter models the probability of obtaining zero. The distribution features of the variable “phoretic Varroa” (*Vp_t_*) require dividing by 100 in order to fit the data to the interval [0, 1]. We then modeled this new response variable by a zero-inflated beta distribution, with parameter variation depending on covariates. A first model selection was performed to choose the best variables to model µ (see AICc comparisons in Table 1; more details are provided in Supplementary Materials Table S1). At the end of this preliminary selection, two models including the apiary random factor were retained, one for the 1-month adjustment and the other for the 3-month adjustment, noted (*) and (**) in Table 1, respectively.

The final models (A and B, see below) were obtained after a second variable selection based on AICc comparisons, using the modeled σ and ν added to the (*) and (**) preliminary models. The number of phoretic Varroa present at *t* was modeled by the following zero-inflated beta models (BEZI in “*gamlss*”):

*Vp_t_* ~ BEZI (µ, σ)   with (1 − ν) probability *Vp_t_* = 0   with ν probability

*For data adjustment at one month*: (A)

µ = logit^−1^ (α_0_ + α_1_*Vb*_t−x_ + α_2_*Cp*_t−x_ + α_3_*D*_t_ + *Ap*)
σ = exp (β_0_ + β_1_*Vb*_t−x_ + β_2_*D*_t_ + *Ap*)
ν = logit^−1^ (γ_0_ + γ_1_*Vb*_t−x_ + γ_2_*Cp*_t−x_ + γ_3_*D*_t_ + *Ap*)

*For data adjustment at three months*: (B)

µ = logit^−1^ (α_0_ + α_1_*Vb*_t−x_ + *Ap*)
σ = exp (β_0_ + *Ap*)
ν = logit^−1^ (γ_0_ + γ_1_*Vb*_t−x_ + γ_2_*Vp*_t−x_)

where the α, β, and γ parameters are coefficients used to model µ, σ, and ν, respectively. As a consequence of this second variable selection, the final AICc was −3179.2 (A) and −343.5 (B) (see details in Tables S2 and S3). Varbrood, which was retained by model selection in all cases except for σ of model B, appeared as the most important explanatory variable.

### 2.2. Goodness of Fit and Prediction Evaluation

#### 2.2.1. Parameter Uncertainty

For models A and B, the α, β, and γ parameters associated with µ, σ, and ν were estimated and their CI_95%_s were computed (see Table 2). Based on the intercept, we can note that varbrood had the largest influence on the data adjustment for each parameter of model A. Moreover, the order of influence of model covariates was the same regardless of the parameter: varbrood > date > capped brood cells. For ν of model B, phoretic Varroa had a larger influence than varbrood. The CI_95_ range as positively correlated with covariate weights, i.e., the greater the weight, the larger the uncertainty.

Moreover, the apiary effect depended on the horizon of prediction. Thus, the mean apiary effect was zero with varying estimated standard deviations depending on the data adjustment; at one month, the estimated standard deviation was 0.285, with a standard deviation of this estimate of 0.798, and at three months, the estimated standard deviation was 0.681, with a standard deviation of this estimate of 0.914 (see Supplementary Materials Tables S2 and S3).

#### 2.2.2. Prediction Quality

The prediction quality can be evaluated using confidence intervals and error rates of models. Table 3 shows that for cross-validation, 97.6% (N = 4999) of sampled phoretic Varroa mites were in their CI_95%_ with model A and 97.3% (N = 2328) with model B. These coverage rates are heterogeneous with respect to *Vp_t_*: they overestimate the targeted values (95%, 70%, or 50%) when *Vp_t_* ≤ 3, they are consistent when 3 < *Vp_t_* ≤ 10, and they are significantly lower than the targeted values when *Vp_t_* > 10, which roughly corresponds to only 5–10% of the hives. These results hold approximately for all tackled cases (cross-validation and training validation; models A and B).

**Figure 1:**
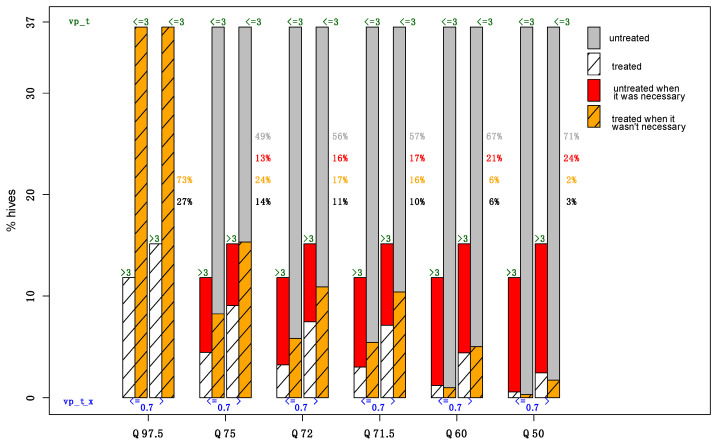
In this figure, 5 scenarios are presented with increasing risk (from left to right) taken by the beekeeper to not treat when the model predicts it was necessary or to treat when it was unnecessary. The risk is inversely proportional to the measure of quantile Q. For each level of risk, four cases are represented: (1) Hives with vp_t_x (i.e., *Vp* at *t = 0*) < = 0.7 and vp_t (i.e., *Vp* three months later) >3; (2) Hives with vp_t_x (i.e., *Vp* at *t = 0*) < = 0.7 and vp_t (i.e., *Vp* three months later) < = 3; (3) Hives with vp_t_x (i.e., *Vp* at *t = 0*)> 0.7 and vp_t (i.e., *Vp* three months later) < = 3; (4) Hives with vp_t_x (i.e., *Vp* at *t = 0*)> 0.7 and vp_t (i.e., *Vp* three months later) < = 3.

Predicted quantiles were used as an indicator of the accuracy of the prediction aimed by the model, i.e., the proportion of hives to be treated against Varroa. Predicting values by simulation may be seen as minimizing the risk of an incorrect prediction (the risk of unnecessarily increasing the number of hives to be treated) or may be necessary to more accurately target the correctly predicted value (the risk of ignoring a proportion of hives which should be treated and which will not be). For model B, outputs are based on the average Varroa load in April of 0.7 phoretic Varroa mites per 100 bees [30] (quoted *Vp*_t−x_) and the threshold of 3 phoretic Varroa mites per 100 bees at the beginning of summer [10] (quoted *Vp_t_*). The model indicates for each colony whether or not to treat (prediction that the threshold will exceed three Varroa mites). Figure 1 describes two extreme situations that correspond to two treatment strategies. The first two strategies, represented by Q97.5 and Q85, are no-risk situations because the model indicated that all colonies are to be treated, and thus no risks are taken of having a colony that exceeds the threshold of three. In these cases, the input costs are great, and 73% of colonies are unnecessarily treated. The second strategy (Q50) is an attempt to justify no treatment, and it estimates the respective risk; it provides reasons not to treat 71% of colonies at the risk of not treating the 24% of colonies that need treatment. This could be seen as the price to pay for engaging in a process of decreasing inputs. Intermediate quantiles allow beekeepers to find correct indicators based on calculated trade-offs. For example, considering indicators for Q72 (or Q71.5), 27% of colonies observed exceed the threshold of three; the model predicted to treat 11% when necessary (10% for Q71.5) and 17% when not necessary (16% for Q71.5). In these cases, there were as many colonies that were treated when not necessary (17%—in orange) as colonies untreated when necessary (16%—in red) for Q72, and the inverse occurred for Q71.5 (Figure 1).

The first and third cases are the hives that are necessary to treat. The percentages of these four categories are provided for each level of risk.

This figure is based partly on Table S4 of Supplementary Materials; Tables S4 and S5 show all results for models A and B of the two model evaluations (cross-validation and training validation). For both models, the smaller the quantile, the lower the global error rate. For larger quantiles (Q97.5 and Q85), models predicted better *Vp_t_* when the phoretic Varroa number exceeded the threshold of three Varroa mites at *t*. Model predictions of *Vp_t_* were relatively good when the earlier phoretic Varroa number was at three, the maximum. However, models failed to produce correct predictions when the mite number at *t-x* was higher than three for model A and higher than 0.7 for model B.

## 3. Discussion

### 3.1. Selected Variables

The Handy VarLoad (HVL) model allowed for the prediction of the Varroa load at a given moment *t*, as a function of the previously observed Varroa load and of the available area for their reproduction, i.e., the number of honey bee brood cells.

Seasons influence the Varroa load, but only in the short term. This could be explained by the fact that, in one month, a beekeeper management intervention or a particular climatic event can have an effect on one or two Varroa generations, as the generation interval of capped brood is 12 days. The mite population growth rate is exponential during short periods (three months) and when mite populations are low; in contrast, Varroa population growth follows a logistic dynamic over longer periods (covering the entire production period) when density-dependent factors influence population growth [12]. Consequently, an event which increases or decreases Varroa reproduction may change the short-term Varroa load but have an insignificant influence on the long-term Varroa load. For example, disruption of honey bee colony broods could be offset by the Varroa population growth itself. Conversely, if colony brood disruption speeds up Varroa reproduction, the mite population size eventually stabilizes due to density dependence [25,31]. Moreover, during a three-month period, colonies undergo a series of favorable and unfavorable disruptions for Varroa development, particularly climatic, which will balance each other out. Finally, the apiary effect acts regardless of the delay between two phoretic Varroa measurements. Thus, the biological variability between colonies, the differences in management strategy between beekeepers, year, and region (climate) influence the Varroa load of the colony [32,33,34,35].

Contrary to previous mathematical models on Varroa load, the HVL model allows one to obtain a prediction with a measure of uncertainty, as well as the associated uncertainty for each parameter. The model uncertainty includes variability at the inter-apiary scale, in beekeeping management strategies, and in year and region effects. The apiary effect included in the model induces a large amount of prediction uncertainty, but, at the same time, it assimilates the sampling diversity related to apiary characteristics (management, year, and region).

### 3.2. Beekeepers’ Interest

The model presented here allows one to have a representation of the risk beekeepers take by not treating the apiary, according to the percentage of colonies that exceed the threshold of three Varroa mites. Different quantiles propose different decision-making indicators for beekeepers taking into account trade-offs between cost, time, and environmental effects of treatments, on the one hand, and the risk of losing infested colonies, on the other.

Moreover, beside economic trade-offs, Varroa treatments are not without consequences and, indeed, may induce acaricide resistance in Varroa [3,4,5], which is why beekeepers should treat only when economic risks are real. It is worth noting that treatments during the beekeeping season are not efficient over the long term [36]. These types of treatment must be used only when the aim is to temporarily decrease the Varroa load to optimize honey flow performance. Thus, this model takes into account integrated pest management.

The model can also be used to determine which apiaries should be given priority on lavender and sunflower fields if the spot number is limited. However, despite the fact that managing colonies at the apiary scale is more efficient, as honey bee colony performances are highly dependent on the characteristics of any apiary (Kretzschmar et al., unpublished data), beekeepers may want to manage Varroa at the colony scale and thus strictly follow the model prediction.

### 3.3. Limits and Prospects of the Model

The choice to use only easy-to-measure variables in the field impairs the model’s goodness of fit and, consequently, the estimation/prediction accuracy. Taking into account other variables (Varroa foundress density, Varroa infestation rate in the capped brood, natural death of Varroa mites measured on sticky boards, etc.) would have allowed better predicting the Varroa load. Including these additional variables in the present model could have easily improved its prediction power. Nevertheless, it would be far too long and complex to collect that type of data in the actual schedule of a beekeeper. If the sampling plan is unrealistic and impracticable at a large scale, the HVL model will be worthless. However, such improved models could be developed for researchers or technicians who work on a smaller scale and need to have better precision in their experimental frameworks. Another limit of this study is the sampled colony number: the more hives sampled, the better the estimation. In the present study, as the number of repetitions for each factor (management, year, and region) is limited, our sampling variation increased model uncertainty. Nevertheless, the Handy VarLoad model will be improved by the accumulation of data issued from the numerous experiments in which the two handy variables it uses (phoretic Varroa load and capped brood area) are commonly collected. As the database on which the model is based increases, the effect of covariates (apiary, region, season, beekeeping practices, etc.) can be better integrated.

## 4. Materials and Methods

### 4.1. Data Sampling

Data were collected from 310 colonies from 2014 to 2016 in three regions of France (PACA, AURA, and Occitanie; “dataset1”) and from 720 colonies in 2018 in three regions of France (PACA, Nouvelle Aquitaine, and Centre; “dataset2”). Most of the colonies were kept on 10-frame Dadant hives and contained hybrid *Apis mellifera* L. queens. Colonies belonged to commercial beekeepers and thus displayed different sizes, dynamics, and management styles, which allowed us to take into account the variability which exists between beekeepers and apiaries. No treatment against Varroa was applied during the sampling periods.

At each sampling point, the amount of capped brood (noted *Cb*) was determined according to the ColEval method [37], and the phoretic mite load was estimated by sampling around three hundred bees (or 45 g) from a frame containing an uncapped brood. Sampled bees were washed with a detergent solution and the number of Varroa mites retrieved (noted *Vp*) was counted [38]. Finally, to take into account seasonality, a “date” variable (noted *D*) was also created in which days were reported on a perpetual calendar with day 1 starting on 15 March of each year. This variable described the number of days ran from an initial time, which corresponds to the beginning of the measurable increase in the Varroa population after wintering. In our case, it corresponded approximately to the middle of March.

Sampling points were repeated at 30-day intervals, except for apiaries R16 to R18 (“dataset1”), in which measurements were sometimes performed every 12 days to mimic the generation interval of capped broods.

### 4.2. Statistical Methods

#### 4.2.1. Distribution Adjustment on “dataset1”

All statistics were performed using the statistical software R version 3.3.0 [39]. Estimation of model parameters was carried out using the “gamlss” function of the eponymous package (Rigby and Stasinopoulos, 2005). The response variable (number of observed Varroa mites per 100 bees) was modeled with a generalized additive model for location, scale, and shape (GAMLSS). GAMLSS is an extension of the generalized linear model and the generalized additive model. It is a distribution-based approach to semiparametric regression models, in which all the parameters of the assumed distribution for the response can be modeled as additive functions of the explanatory variables, such as the *location* (e.g., *mean* µ), the *scale* (e.g., variance σ^2^), the *shape* (skewness and kurtosis), and some *inflation* (e.g., at zero, ν). Moreover, we chose to use GAMLSS because it offers numerous choices for the distribution of the response variable and is suitable for time series data (Rigby and Stasinopoulos, 2001). GAMLSS was fitted to data using maximum (penalized) likelihood estimation implemented with the RS algorithm, which does not require accurate starting values for µ, σ, and ν to ensure convergence in comparison with the CG algorithm [40,41]. The most parsimonious model with the lowest corrected Akaike’s information criterion (AICc) [42], was selected; models with differences in AICc values lower than or equal to two were considered to be equivalent. We chose this selection criterion because, it is the most suitable criterion to model selection in predictive models for ecology and time series applications including forecasting [43]. Thus, it allows for the selection of the model that will best predict the response variable, i.e., the model with the best predictive accuracy.

Variables, which were described above, were transformed as follows to comply with the scaling conditions during model fitting:

(1) $$Cb=\frac{Cb0}{100}$$

(2) $$Vp=\frac{Vp0*100*0.14}{sw}$$

(3) $$Vb=\log\left(\frac{Vp}{Cb+130}*100+1\right)*50$$

where *Cb* (Equation (1)) is a scaled value of the number of capped brood cells *Cb*0; *Vp* (Equation (2)) is the normalized rational number of Varroa mites for 100 honey bees (called “phoretic Varroa” in the present study), knowing that the weight per bee is 0.14 g, and *sw* in Equation (2) is the sampling weight of bees; *Vb* is a variable called “varbrood”, built to take into account the role of the amount of brood in the regulation of Varroa reproduction, and, more specifically, to integrate the fact that the more spread out the capped brood, the harder it is to capture phoretic Varroa mites hidden in the capped brood. The varbrood variable was thus obtained by taking the Neperian logarithm of the number of phoretic Varroa and dividing it by the number of capped brood cells. In Equation (3), 130 corresponds to the *Cb* median, 100 and 50 multipliers are necessary for the scale, and +1 is used to avoid obtaining log(0). These three quantitative variables were mathematically reduced to the same scale, in order to be able to compare their respective weights during model adjustment. The date (measured as a number of days after the first measurement) was used without transformation.

The rational number of phoretic Varroa mites present at *t* (*Vp_t_)* was modeled in the GAMLSS framework by a zero-inflated beta distribution with mean µ, standard deviation σ, and inflation at zero ν. Different specifications for µ, σ, and ν were used (see Results section). Our models were designed to predict *Vp_t_* from explanatory variables typically collected at time *t−x*. Two horizons of prediction *x* were considered: a short-term horizon (*x* = 1 month, noted model A hereafter) and a long-term horizon (*x* = 3 months, noted model B hereafter). For *x* = 1 (model A), all data were used to fit the models (867 observations), whereas for *x* = 3 (model B), all the data providing this interval were used to avoid the use of time-overlapping pairs of observations (93 observations). Phoretic Varroa numbers, capped brood cell numbers, and varbrood present at *t−x*, as well as the date at *t*, were exploited as fixed factors; they are denoted by *Vp*_t−x_, *Cb*_t−x_, *Vb*_t−x_, and *D*_t_, respectively. Moreover, an «apiary» factor (noted *Ap*) was used as a random factor and includes the variability of the apiary, beekeeping management strategy, and year and region effects.

#### 4.2.2. Goodness of Fit and Prediction Including “dataset2”

To assess the goodness of fit of the selected models, we explored the uncertainty of parameters and the prediction quality by comparing the predicted and observed values of *Vp_t_*. We evaluated the prediction quality using two methods: cross-validation and training validation. In both methods, model performance was evaluated on data not included in the sample used to estimate model parameters.

For increasing the domain where the uncertainty of the parameters and the prediction quality of the models could be explored, a larger dataset (“dataset2”) was added to the first dataset (“dataset1”) with which the model parameters were estimated.

In the cross-validation method, observations of the hives of a given apiary were removed from the database, the model was fitted to the remaining data, and estimated parameters were plugged in to predict *Vp_t_* for the hives of the apiary whose observations were removed (this case corresponds to predicting *Vp_t_* for a new apiary based on observations collected from other apiaries). This procedure was repeated for each apiary of the dataset (i.e., 54 times for model A and 40 times for model B) and allowed us to provide averaged cross-validation assessments of the prediction performance.

The prediction performance was assessed with respect to the two following criteria:

- The actual coverage of 95%, 70%, and 50% confidence intervals of *Vp_t_* (denoted by CI_95%,_ CI_70%_, and CI_50%_), providing the proportion of times that the true value of *Vp_t_* is contained within the CI;
- The use of different predicted quantiles of *Vp_t_* (namely, Q_97,5%_, Q_85%_, Q_75%_, and Q_50%_) to evaluate the risk that the actual *Vp_t_* exceeds the problematic threshold of 3 Varroa mites for 100 bees.

These criteria (CI and quantiles) were empirically calculated from 1000 simulations of the zero-inflated beta distribution in which the estimated values of µ, σ, and ν were inserted (random factors incorporated into µ, σ, and ν were randomly drawn at each simulation from centered normal distributions with standard deviations equal to their estimated values). Note that estimation uncertainty was neglected in this simulation procedure; this choice may lead to un-calibrated confidence intervals and quantiles. The comparisons of quantiles Q_97.5%_, Q_85%_, Q_75%_, and Q_50%_ with the threshold of 3 Varroa mites for 100 bees can be used as indicators to assess whether *Vp_t_* will exceed this problematic threshold. The efficiency of these indicators was assessed with the error rate $\tau^{error}\left(\alpha \right)$ calculated as the ratio between (i) the number of hives for which the predicted quantile Q_α_(*Vp_t_*) is less than or equal to 3 at time *t*, whereas the actual observation *Vp_t_* is greater than 3, and (ii) the number of observations *Vp_t_* greater than 3:

(4) $$\tau^{error}\left(\alpha \right)=\frac{\sum_{R=1}^{K}\sum_{r=1}^{N_{R}}1\left(\;Vp_{t,R,r}>3,\;Q_{\alpha }^{-R}\left(\;Vp_{t,R,r}\right)\leq 3\right)}{\sum_{R=1}^{K}\sum_{r=1}^{N_{R}}1\left(\;Vp_{t,R,r}>3\right)},$$

where *K* is the number of apiaries (54 for model A and 40 for model B), $N_{R}$ is the number of hives in the apiary *R* (which ranges between 7 and 51), *Vp_t_*_,*R*,*r*_ is the observed value of phoretic Varroa at time *t* for the hive *r* of the apiary *R*, and $Q_{\alpha }^{-R}\left(\;Vp_{t,R,r}\right)$ is the predicted quantile at α% of *Vp_t,R,r_* for the hives of the apiary *R* whose observations were removed (−*R*). The indicator function E→**1I** takes the value of 1 if event E is true, or otherwise 0.

Equation (4) presents the case in which observations are greater than 3 and predictions are less than or equal to 3, and $\tau^{error}\left(\alpha \right)$ was also calculated when observations are less than or equal to 3 and predictions are greater than 3. The error rate $\tau^{error}\left(\alpha \right)$ can be computed in other specific conditions, for example, conditions related to the number of phoretic Varroa at *t−*x (*Vp_t–x_*_,*R*,*r*_).

In the training validation method, observations of the hives of all apiaries after a specific date, *t_A_* = 31 (15 April) for model A, and *t_B_* = 118 (11 July) for model B, were removed from the database, the model was fitted to remaining data, and estimated parameters were plugged in to predict *Vp_t_* for the hives of all apiaries after *t_A_* or *t_B_* (this case corresponds to predicting *Vp_t_* for an apiary already installed, based on observations collected beforehand from this apiary). This procedure was repeated for each year of the dataset (i.e., from 2014 to 2016 and 2018 for both models A and B) and allowed us to provide averaged training validation assessments of the prediction performance already introduced in the cross-validation approach.

## Figures and Tables

**Table 1:** Comparisons of the tested models investigating the influence of phoretic Varroa numbers (per 100 bees) at *t* = 0, capped brood cell numbers, varbrood, and date of predicted phoretic Varroa numbers as a function of the estimation length, using the AICc criterion. N = 867 for data adjustment at one month (x = 1) and N = 93 for data adjustment at three months (x = 3).

	Adjustment for x = 1	Adjustment for x = 3
Model	AICc	AICc
phoretic Varroa	−2477.5	−268.3
capped brood cells	−2320.6	−261.8
varbrood (**)	−2540.1	**−296.9**
date	−2413.0	−260.9
phoretic Varroa + capped brood cells	−2488.0	−271.1
phoretic Varroa + date	−2561.7	−266.3
phoretic Varroa + varbrood	−2538.2	−295.7
capped brood cells + date	−2412.1	−261.4
capped brood cells + varbrood	−2580.1	−297.7
date + varbrood	−2618.2	−294.7
phoretic Varroa + capped brood cells + date	−2564.9	−269.1
phoretic Varroa + capped brood cells + varbrood	−2582.4	−296.0
phoretic Varroa + date + varbrood	−2616.2	−293.8
capped brood cells + date + varbrood (*)	**−2645.5**	−295.5
phoretic Varroa + capped brood cells + varbrood + date	−2647.8	−293.9
(*) and (**) + apiary random effect	**−2651.1**	**−316.9**

**Table 2:** Estimated coefficient and 95% confidence interval (CI_95%_) of models A and B investigating the influence of varbrood, capped brood cells, phoretic Varroa, and date on the number of phoretic Varroa mites for mu, sigma, and nu parameters.

Model	Parameter	Covariate	Estimated Coefficient	Lower 95% CI	Upper 95% CI
A	Mu	Intercept	−5.830	−6.021	−5.640
		varbrood	0.025	0.021	0.028
		capped brood cells	0.002	0.001	0.003
		date	0.014	0.012	0.015
	Sigma	Intercept	6.579	6.233	6.925
		varbrood	−0.023	−0.030	−0.016
		date	−0.018	−0.021	−0.015
	Nu	Intercept	2.073	1.475	2.672
		varbrood	−0.063	−0.087	−0.039
		capped brood cells	−0.003	−0.006	−0.001
		date	−0.032	−0.039	−0.025
B	Mu	Intercept	−3.982	−4.175	−3.790
		varbrood	0.023	0.019	0.027
	Sigma	Intercept	4.460	4.140	4.779
	Nu	Intercept	−0.701	−1.468	0.065
		varbrood	−0.077	−0.167	0.012
		phoretic Varroa	−3.786	−10.747	3.176

**Table 3:** Coverage rates of confidence intervals (CI_95%,_ CI_70%_, and CI_50%_) of *Vp_t_* for both approaches, cross-validation and training validation, for models A and B. The coverage rate provides the proportion of times that the CI contains the true value of *Vp_t_*. For each method and each model, numbers of observed hives are reported for each class of *Vp_t_*.

**Cross-Validation**									
**Model A**	**Model B**	**Observed *Vp_t_***	**Model A**			**Model B**			
**Observed Colony Numbers**			**CI_95%_**	**CI_70%_**	**CI_50%_**	**CI_95%_**	**CI_70%_**		**CI_50%_**
4999	2328	all	97.6	83.6	67.7	97.3	83.1		67.8
4027	1700	≤3	99.6	91.5	76.3	99.7	97.9		87.9
724	526	>3 and ≤10	92.7	53.7	34.5	99.8	51.1		16
248	102	>10	80.6	42.3	24.2	45.1	2		0
**Training Validation**									
**Model A**	**Model B**	**Observed *Vp_t_***	**Model A**			**Model B**			
**Observed Colony Numbers**			**CI_95%_**	**CI_70%_**	**CI_50%_**	**CI_95%_**		**CI_70%_**	**CI_50%_**
1438	749	all	92.6	75.3	61.8	57.8		39	26
1140	546	≤3	95.9	82.6	69	61.2		44.1	29.9
229	137	>3 and ≤10	82.1	49.8	37.6	60.6		29.9	17.5
69	66	>10	72.5	39.1	21.7	24.2		15.2	12.1

## Data Availability

All data are available upon request to the corresponding author.

## Supplementary Materials

The following are available online at https://www.mdpi.com/article/10.3390/pathogens10060678/s1, Table S1: Comparisons of the tested models investigating the influence of phoretic Varroa mite (per 100 bees) numbers, capped brood cell numbers, varbrood, and date on next phoretic Varroa numbers as a function of the estimation length, using the AICc criterion. N = 867 for data adjustment at one month (x = 1) and N = 93 for data adjustment at three months (x = 3), Table S2: R graphic output of model A summary, Table S3: R graphic output of model B summary, Table S4: Performance comparisons between different quantiles (Q97.5, Q85, Q75, Q50) for models A and B, depending on the observed phoretic Varroa numbers at t−x and the observed phoretic Varroa numbers at *t*. Error rates represent the colony percentage for which the Varroa load at the horizon of prediction was badly predicted with the cross-validation method. For each quantile, the number of hives to treat depends on the error rate for which their percentages were reported, Table S5: Performance comparisons between different quantiles (Q97.5, Q85, Q75, Q50) for models A and B, depending on the observed phoretic Varroa numbers at t-x and the observed phoretic Varroa numbers at t. Error rates represent the colony percentage for which the Varroa load at the horizon of prediction was badly predicted with the training validation method. For each quantile, the number of hives to treat depends on the error rate for which their percentages were reported.
