# Supplementary material for: To Treat or Not to Treat Bees? Handy VarLoad: A Predictive Model for Varroa destructor Load
Source: Pathogens. 2021 May 30;10(6):678. doi: 10.3390/pathogens10060678 (PMC8229881; doi:10.3390/pathogens10060678)
Supplement: Supplementary file 1 [file pathogens-10-00678-s001.zip › pathogens-1211831-supplementary.pdf]

SUPPLEMENTARY MATERIALS

Table S1. Comparisons of the tested models investigating the influence of phoretic Varroa (per 100 bees) numbers, capped brood cell numbers, varbrood and date on next phoretic Varroa numbers in function of the estimation length, using the AICc criterion. N=867 for a data adjustment at one month (x=1) and N=93 for a data adjustment at three months (x=3).

	Adjustment for x=1		Adjustment for x=3	
	no random effect	*apiary random effect*	*no random effect*	*apiary random effect*
**Models**	**AICc**	**AICc**	**AICc**	**AICc**
phoretic Varroa	-2477.5	-2487.4	-268.3	-284.0
capped brood cells	-2320.5	-2356.8	-261.8	-282.7
varbrood	-2540.1	-2539.6	-296.9	**-316.9**
time	-2413.0	-2505.7	-260.9	-280.6
phoretic Varroa + capped brood cells	-2488.0	-2493.4	-271.1	-285.4
phoretic Varroa + time	-2561.7	-2599.9	-266.3	-281.6
phoretic Varroa + varbrood	-2538.2	-2537.6	-295.7	-316.5
capped brood cells + time	-2412.1	-2505.2	-261.4	-280.4
capped brood cells + varbrood	-2580.1	-2578.8	-297.7	-316.1
time + varbrood	-2618.2	-2635.2	-294.7	-314.6
phoretic Varroa + capped brood cells + time	-2564.9	-2598.2	-269.1	-282.8
phoretic Varroa + capped brood cells + varbrood	-2582.4	-2583.1	-296.0	-314.7
phoretic Varroa + time + varbrood	-2616.2	-2633.1	-293.8	-313.9
**capped brood cells + time + varbrood**	-2645.5	**-2651.1**	-295.5	-313.6
phoretic Varroa + capped brood cells + varbrood + time	-2647.8	-2652.7	-293.9	-312.0

Table S2. R graphic output of model A summary.

```
*******************************************************************
Family:  c("BEZI", "Zero Inflated Beta")

Call:  gamlss(formula = vp_t/100 ~ cf_t_x + t + calc + re(random = ~1 |
    rucher), sigma.formula = ~1 + calc + t + re(random = ~1 |
    rucher), nu.formula = ~1 + calc + t + cf_t_x +
    re(random = ~1 | rucher), family = BEZI, data = od)

Fitting method: RS()

-------------------------------------------------------------------
Mu link function:  logit
Mu Coefficients:
              Estimate Std. Error t value Pr(>|t|)
(Intercept) -5.8302230  0.0972868 -59.928  < 2e-16 ***
cf_t_x       0.0018841  0.0004142   4.549 6.23e-06 ***
t            0.0135256  0.0008812  15.350  < 2e-16 ***
calc         0.0247792  0.0017341  14.290  < 2e-16 ***
---
Signif. codes:  0 '***' 0.001 '**' 0.01 '*' 0.05 '.' 0.1 ' ' 1

-------------------------------------------------------------------
Sigma link function:  log
Sigma Coefficients:
             Estimate Std. Error t value Pr(>|t|)
(Intercept)  6.579100   0.176707  37.232  < 2e-16 ***
calc        -0.023107   0.003468  -6.664 4.94e-11 ***
t           -0.017826   0.001686 -10.576  < 2e-16 ***
---
Signif. codes:  0 '***' 0.001 '**' 0.01 '*' 0.05 '.' 0.1 ' ' 1

-------------------------------------------------------------------
Nu link function:  logit
Nu Coefficients:
             Estimate Std. Error t value Pr(>|t|)
(Intercept)  2.073460   0.305377   6.790 2.17e-11 ***
calc        -0.062737   0.012270  -5.113 3.96e-07 ***
t           -0.032335   0.003516  -9.197  < 2e-16 ***
cf_t_x      -0.003014   0.001841  -1.637    0.102
---
Signif. codes:  0 '***' 0.001 '**' 0.01 '*' 0.05 '.' 0.1 ' ' 1

------------------------------------------------------------------
NOTE: Additive smoothing terms exist in the formulas:
 i) Std. Error for smoothers are for the linear effect only.
ii) Std. Error for the linear terms maybe are not accurate.
------------------------------------------------------------------
No. of observations in the fit:  867
Degrees of Freedom for the fit:  58.69432
      Residual Deg. of Freedom:  808.3057
                      at cycle:  20

Global Deviance:     -3305.293
            AIC:     -3187.904
            SBC:     -2908.223
******************************************************************
> AICc(model)
[1] -3179.224
> model$mu.coefSmo
[[1]]
Linear mixed-effects model fit by maximum likelihood
  Data: Data
  Log-likelihood: -5547.429
  Fixed: fix.formula
(Intercept)
-0.09407978

Random effects:
 Formula: ~1 | rucher
        (Intercept)  Residual
StdDev:   0.2845794 0.7978971

Variance function:
 Structure: fixed weights
 Formula: ~W.var
Number of Observations: 867
Number of Groups: 19
```

Table S3. R graphic output of model B summary.

```
******************************************************************
Family:  c("BEZI", "Zero Inflated Beta")

Call:  gamlss(formula = vp_t/100 ~ calc + re(random = ~1 |      rucher), sigma.formu
la = ~1 + re(random = ~1 |
    rucher), nu.formula = ~1 + calc + vp_t_x, family = BEZI,      data = classe)

Fitting method: RS()

------------------------------------------------------------------
Mu link function:  logit
Mu Coefficients:
             Estimate Std. Error t value Pr(>|t|)
(Intercept) -3.982359   0.098289  -40.52   <2e-16 ***
calc         0.023025   0.001851   12.44   <2e-16 ***
---
Signif. codes:  0 '***' 0.001 '**' 0.01 '*' 0.05 '.' 0.1 ' ' 1

------------------------------------------------------------------
Sigma link function:  log
Sigma Coefficients:
            Estimate Std. Error t value Pr(>|t|)
(Intercept)   4.4595     0.1632   27.32   <2e-16 ***
---
Signif. codes:  0 '***' 0.001 '**' 0.01 '*' 0.05 '.' 0.1 ' ' 1

------------------------------------------------------------------

Nu link function:  logit
Nu Coefficients:
            Estimate Std. Error t value Pr(>|t|)
(Intercept) -0.70140    0.39117  -1.793   0.0770 .
calc        -0.07715    0.04573  -1.687   0.0958 .
vp_t_x      -3.78571    3.55184  -1.066   0.2899
---
Signif. codes:  0 '***' 0.001 '**' 0.01 '*' 0.05 '.' 0.1 ' ' 1

------------------------------------------------------------------
NOTE: Additive smoothing terms exist in the formulas:
 i) Std. Error for smoothers are for the linear effect only.
ii) Std. Error for the linear terms maybe are not accurate.
------------------------------------------------------------------
No. of observations in the fit:  93
Degrees of Freedom for the fit:  18.81129
      Residual Deg. of Freedom:  74.18871
                      at cycle:  20

Global Deviance:     -391.2824
            AIC:     -353.6598
            SBC:     -306.0183
******************************************************************
> AICc(model)
[1] -343.4758

> model$mu.coefSmo
[[1]]
Linear mixed-effects model fit by maximum likelihood
  Data: Data
  Log-likelihood: -378.1895
  Fixed: fix.formula
(Intercept)
 -0.2312673

Random effects:
 Formula: ~1 | rucher
        (Intercept)  Residual
StdDev:     0.68064 0.9136619

Variance function:
 Structure: fixed weights
 Formula: ~W.var
Number of Observations: 93
Number of Groups: 8
```

Table S4. Performance comparisons between different quantiles (Q97.5, Q85, Q75, Q50) for models A and B, depending on the observed phoretic Varroa numbers at t-x and the observed phoretic Varroa numbers at t. Error rates represent the colony percentage, which were badly predicted with cross-validation method to predict new apiary. For each quantile, number of hives to treat depends of error rate and their percentage were reported.

Cross-validation

Model A

**Observed colony numbers**	**Observed Vp_{t-x}**	**Observed Vp_t**	**Q 97.5**		**Q 85**		**Q 75**		**Median**	
			**Error rate (%)**	**Number of hives to treat**	**Error rate (%)**	**Number of hives to treat**	**Error rate (%)**	**Number of hives to treat**	**Error rate (%)**	**Number of hives to treat**
584	≤3	>3	2.1	572 (97.9%)	19.9	468 (80.1%)	39.6	353 (60.4%)	77.2	133 (22.8%)
3445	≤3	≤3	65.4	2253 (65.4%)	26.9	927 (26.9%)	14.1	486 (14.1%)	2.5	86 (2.5%)
388	>3	>3	0	388 (100%)	3.1	376 (96.9%)	12.9	338 (87.1%)	51.3	189 (48.7%)
582	>3	≤3	98.6	574 (98.6%)	80.4	468 (80.4%)	62.4	363 (62.4%)	19.9	116 (19.9%)
4999	All	All	56.8	3787 (75.8%)	30.5	2239 (44.8%)	22.6	1540 (30.8%)	13	524 (10.5%)

Model B

**Observed colony numbers**	**Observed Vp_{t-x}**	**Observed Vp_t**	**Q 97.5**		**Q 85**		**Q 75**		**Median**	
			**Error rate (%)**	**Number of hives to treat**	**Error rate (%)**	**Number of hives to treat**	**Error rate (%)**	**Number of hives to treat**	**Error rate (%)**	**Number of hives to treat**
266	≤0.7	>3	0	266 (100%)	0	266 (100%)	60.5	105 (39.5%)	95.5	12 (4.5%)
858	≤0.7	≤3	100	858 (100%)	100	858 (100%)	23.8	204 (23.8%)	1.5	13 (1.5%)
368	>0.7	>3	0	368 (100%)	0	368 (100%)	38	228 (62%)	84.5	57 (15.5%)
836	>0.7	≤3	100	836 (100%)	100	836 (100%)	39.1	327 (39.1%)	4.2	35 (4.2%)
2328	All	All	72.8	2328 (100%)	72.8	2328 (100%)	35.7	864 (37.1%)	26.3	117 (5%)

Example: for model A and Q97.5, colonies, that had less or equal to 3 Varroa one month before and that now have more than 3 varroa, were predicted with an error rate of 2.1%. Thus, 572 colonies of 584 colonies (fulfilled threshold condition above) must be treated according to the model (i.e. 97.9% of the 584 hives). Global error for this quantile is 56.8% with 3787 (572+2253+388+574) colonies to treat (75.8% of 4999 colonies), knowing that 2827 (2253+574) colonies will be treat while it's not necessary because they don't exceed the threshold of 3 varroas.

Table S5. Performance comparisons between different quantiles (Q97.5, Q85, Q75, Q50) for models A and B, depending on the observed phoretic Varroa numbers at t-x and the observed phoretic Varroa numbers at t. Error rates represent the colony percentage, which were badly predicted with training-validation method to predict new apiary. For each quantile, number of hives to treat depends of error rate and their percentage were reported.

Training-validation

Model A

Observed colony numbers	Observed Vp_{t-x}	Observed Vp_t	Q 97.5		Q 85		Q 75		Median	
			Error rate (%)	Number of hives to treat	Error rate (%)	Number of hives to treat	Error rate (%)	Number of hives to treat	Error rate (%)	Number of hives to treat
210	≤3	>3	1.9	206 (98.1%)	22.4	163 (77,6%)	32.4	142 (67.6%)	58.1	88 (41.9%)
1086	≤3	≤3	50	543 (50%)	24.2	263 (24.2%)	16	174 (16%)	5.3	58 (5.3%)
88	>3	>3	0	88 (100%)	0	88 (100%)	1.1	87 (98.9%)	2.3	86 (97.7%)
54	>3	≤3	100	54 (100%)	100	54 (100%)	100	54 (100%)	96.3	52 (96.3%)
1438	All	All	41.8	891 (62%)	25.3	568 (39.5%)	20.7	457 (31.8%)	11.7	284 (19.7%)

Model B

Observed colony numbers	Observed Vp_{t-x}	Observed Vp_t	Q 97.5		Q 85		Q 75		Median	
			Error rate (%)	Number of hives to treat	Error rate (%)	Number of hives to treat	Error rate (%)	Number of hives to treat	Error rate (%)	Number of hives to treat
145	≤0.7	>3	8.3	133 (91.7%)	36.6	92 (63.4%)	49.7	73 (50.3%)	71	42 (29%)
397	≤0.7	≤3	45.6	181 (45.6%)	31.7	126 (31.7%)	28.2	112 (28.2%)	20.4	81 (20.4%)
58	>0.7	>3	0	58 (100%)	3.4	56 (96.6%)	13.8	50 (86.2%)	31	40 (69%)
149	>0.7	≤3	88.6	132 (88.6%)	87.9	131 (87.9%)	83.9	125 (83.9%)	63.8	95 (63.8%)
749	All	All	43.4	504 (67.3%)	41.7	405 (54.1%)	42.3	360 (48.1%)	39.7	258 (34.4%)
